# Public health workforce management during a major organizational reform in the World Health Organization African Region Country Offices

**DOI:** 10.3389/fpubh.2025.1562732

**Published:** 2025-07-28

**Authors:** Egide Rwamatwara, Abdulmumini Usman, Ndoungou Salla Ba, Patrick Kabore, Hyelni Kulausa, Olushayo Oluseun Olu, Alex Gasasira, Joseph Cabore, Matshidiso Moeti

**Affiliations:** World Health Organization African Regional Office, Brazzaville, Republic of Congo

**Keywords:** transformation agenda, functional review, people management, managing workforce, country office, WHO African region transformation agenda, WHO African region

## Abstract

Following her appointment as Regional Director of the World Health Organization African Region in February 2015, Dr. Matshidiso Moeti launched the Transformation Agenda to reform and align the organization’s work with regional public health needs. This initiative was prompted by the weaknesses observed during the 2013–2014 Ebola virus disease outbreak in West Africa, particularly delayed response and limited availability of skilled personnel for rapid detection and management. A key component of the agenda was the functional review of the 47 World Health Organization country offices in the region, aimed at optimizing structures and staffing to be fit-for-purpose. Conducted between 2017 and 2023, the functional review sought to tailor the country office capacities to the specific health priorities of all 47 Member States and introduce more effective operational models. This paper examines the management of human resources throughout the functional review process, with a focus on workforce challenges, including anxiety, stress, and uncertainty and the strategies adopted by organization to mitigate their impact. It also reviews the steps taken to strengthen country offices human resource capacities in line with the functional review objectives and draws attention to the challenges, good practices, and lessons learned in managing organizational change during institutional reform.

## Introduction

The Transformation Agenda (TA) of WHO in the African region sought to engender a regional WHO secretariat and country offices appropriately resourced and equipped to deliver on the organizational mandate; “an organization that meets the needs and expectations of its stakeholders” (1). It aimed to make WHO a more transparent, responsive and results driven organization, and capable of meeting stakeholders’ expectations (1). Additionally, it was geared toward stimulating regional health through four focus areas, namely, “pro-results values, smart technical focus, responsive strategic operations, and effective communications and partnerships” (2). The new organizational vision aimed at designing core predictable country presence which is aligned with national needs and priorities to improve lives and health for all, including preparing, detecting, and responding to public health emergencies (3). One of the foundations of the TA was a functional review (FR) aimed at transforming the 47 WHO Country Offices in the African Region (WCOs) into entities equipped with structures and adequate staffing that are fit-for-purpose for efficient delivery of WHO mandate as expected by its Member States (4). As such, FR strove to equip WCOs with a workforce with skill-mix and the necessary competencies to adequately address the health needs and priorities of host countries (25, 26). According to Goleman (5), health institutions can be more effective and efficient in delivering their mandate through a business strategy of organizational transformation.

The FR review also considered the support functions and operational processes aiming at making them more responsive and efficient (1, 17). Goleman (5) argues that organizational transformation entails a robust structural reform, including change in organizational design, culture change, the way employees interact among themselves and with partners, delivering quality products and services to sustain competition and to maintain their relevance. A change in organizational culture would be required to consolidate both strategic, structural and organizational operational changes (6, 16, 18).

The FR was conducted from 2017 to 2019, and the results of the review were implemented from 2019 to 2023. Needless to say, that the new structures which emanated from the FR were not fully staffed and continue to be implemented as and when resources are mobilized. Their implementation required a huge financial capacity that most WCOs did not have then. Hence the implementation and staffing of the new structures was processed in phases through a prioritization of core functions and critical positions for which funding was available.

However, despite its good intentions, namely, to equip WCOs with a fit-for-purpose workforce with required skilled, the FR raised several issues and challenges among the workforce across the 47 WCOs. First, its announcement raised anxiety among the workforce due to the associated uncertainty and the possibility of job insecurity. The concept of functional review aiming to equip WCOs with the right skills was misinterpreted in the general impression was that it would result a massive lay-off of staff. Despite several communications to explain and clarify the purpose and objectives of the FR, the anxiety and associated stress persisted.

The impact of the FR and its implementation on the workforce is the purpose of this paper. It discusses the experiences of the workforce before, during and after the review and the responses of WHO African Regional Office (WHO/AFRO) management toward these challenges.

### The functional review of WHO in the African region

The FR is acclaimed for its originality and its boldness in introducing radical transformation in organizational design, staffing modeling, agile workforce, and new ways of doing business. A paradigm shift in workforce planning and management, in particular, people management during a robust restructuring constitutes some of the biggest lessons learned and good practice of FR (19, 20). The FR mid-term review conducted in 2018 noted that the purpose, the scope and the guideline package of the functional review was comprehensive and that communication prior to the review process was effective (7). It however indicated that communication during and after the functional review could have been more frequent to adequately update staff; timely present the FR outcome and clearly indicate the next steps. It further indicated that career counseling sessions and stress management services were much appreciated by all interviewees. However, it recommended that the WHO/AFRO management should invest more in communication to allay fear among staff.

### The functional review process

The FR started by defining the scope, the purpose, the terms of reference, the expected results and the guidelines for conducting the review and implementing the results. This was to ensure transparency, objectivity and accountability that the TA advocated for from the outset. A team of experts from the technical, operational and human resources management unit of the organization was put in place to conduct the review. Career counseling and stress management services were developed to cushion the effect of the review.

Stakeholders from all 47 Member States of the WHO African Region (WHO/AFR) including government officials, other UN agencies, WHO staff and other partners were consulted to identify the critical needs and priority functions that WHO has a comparative advantage to fulfil for an effective support to the country (4).

The functional review team further translated the country needs and priorities into an ideal structure reflecting the health situation of the country. The next step was to conduct a desk review and skill inventory aiming to determine the required skill-mix to satisfy the identified needs and priorities. The team further reviewed the skills readily available in the country and proceeded to establish the skills gap between the existing and the required skills to make recommendations on how to bridge the gap. The recommendations were formulated into a report submitted to the Regional Director (RD) through the WHO Representative (WR) for consideration, approval and implementation. A townhall meeting was organized for each WCO to give feedback to staff about the findings and the next steps including the process of implementing the results. This was the critical step as it highlighted the procedure to be followed to address functions found to be redundant and what to do with the occupants of the concerned positions. Furthermore, clarifications were provided on how to manage cases where a position has been upgraded or downgraded. Staff were given a chance to ask questions and clarifications pertaining to their specific situations.

### Implementation of the results of functional review

The FR implementation involved staffing newly established human resources structures by filling fixed-term positions for which funding had been secured. Due to widespread budget constraints across the WCOs, not all positions could be funded initially. As a result, WRs were advised to prioritize core positions, while remaining vacancies would be filled as resources became available through ongoing mobilization efforts. Staff holding longer term contracts were reviewed and matched against newly created funded positions. The FR team proposed the matchings in consultation with WRs, which were then submitted to the RD and reviewed by an independent placement committee (PC). This committee, established by the RD, ensured objectivity and compliance with WHO rules. Key principles guiding the process included matching staff to positions of the same grade or one grade lower, and ensuring no promotion occurred without competition.

The matching process resulted in three possible outcomes: single matching where one staff member matched to one position, leading to reassignment; multiple match in which several staff matched to one or fewer positions, prompting internal competition and no matching in which– either a staff member was unmatched (resulting in redundancy and separation), or a position lacked suitable candidates and was opened for recruitment, with priority given to unmatched internal staff.

Clear reasons for non-matching were documented and communicated to staff. Town hall meetings were held in each WCO to explain the outcomes and allow staff to raise concerns. Despite efforts to maintain transparency, objectivity, and fairness, the process generated widespread anxiety, uncertainty, and demotivation among staff. Many staff perceived the FR as a covert downsizing exercise, despite repeated assurances from leadership, particularly the RD that the objective was for organizational strengthening and career development. This perception created resistance, skepticism, and dissatisfaction, especially among those whose positions were abolished or who had anticipated automatic promotions based on past performance. As per FR policy, promotions could only occur through open competition.

Nevertheless, the FR did present career opportunities; over 450 staff were promoted between 2019 and 2023 through competitive processes. Still, the psychological impact and residual concerns among the workforce persisted beyond the formal conclusion of the review.

### Outcome of the functional review

The FR resulted in an ideal structure for each WCO in line with its health situation, needs and priorities (8). The new structures stemming from the FR provided for core positions which if they were fully filled would equip the WCOs with a fit-for-purpose staffing with skill-mix capable to provide adequate support to the host country (22, 23). However, the staffing of these structures required funding which was not readily available in most of the WCOs if not all of them. As such, implementation of the outcome of the FR was executed by prioritizing core functions in line with available funds. In this regard, positions which were not funded could not be staffed. Furthermore, the FR highlighted positions which were redundant and were to be abolished. The profiles of the occupants of these positions were reviewed with the aim of finding positions for which they could match. The staff whose profiles could not match any funded position in the new structure were notified of the abolition of their redundant position and the process of separation was undertaken. The process of staff profile review and matching proved to be more stressful and a source of anxiety among the workforce.

Furthermore, to address the shortage of certain expertise at the WCO level due to limited financial resources, the Multi-country Assignment Teams (MCAT) were introduced. The MCAT was an innovation which consisted of co-locating a group of experts from which they could provide technical support to a group of countries. An MCAT consists of a group of experts positioned in one location and providing technical expertise to 3 or 4 WCOs (9). This approach was considered as an innovative way of doing business by providing technical support to countries with the limited available resources, producing more results with less resources (10). Noting that many WCOs would not mobilize sufficient resources to recruit international experts, the MCAT proved to be an excellent innovative and cost-effective solution. MCATs were initially designed with six (6) identified priority areas each, namely, Reproductive Maternal Neonatal Child and Adolescent Health (RMNCAH); HIV/TB/Hepatitis (HTH); Tropical and Vector Borne Diseases (TVD); Non-Communicable Diseases Control (NCDs); Health Financing (HFI) and Nutrition (NUT). Additional two (2) areas were introduced, namely, Integrated Service Delivery (ISD) and Laboratory Services (LAB). In total 11 locations were initially identified to host a total of 66 experts to intervene in 41 WCOs that could not afford the cost of each individual expertise in those identified core functions. With the introduction of 2 additional functions the number of experts to be located at MCATs rose to 80 located in the various 11 locations. The big five (5) WCOs, namely, Nigeria, Democratic Republic of Congo, Ethiopia, South Sudan and Central African Republic are not part of MCAT as they have pronounced needs requiring individual expertise located in each country. The Republic of Congo is also not part of MCAT approach due to its comparative advantage of hosting WHO African regional office and can directly tap from the expertise of the technical programs hosted there. The implementation of MCATs started in 2021 through the reprofiling the staff who were playing similar roles at Intercountry Support Team (IST) in Burkina Faso, Gabon and Zimbabwe. A total of 22 IST staff were relocated to the various MCAT locations.

However, it is important to note that the process of creating and implementing the MCATs brought with it another challenge. It created a new form of stress and anxiety among the staff designated to move from ISTs to MCATs and among national professionals officers (NPOs) who were playing these roles at WCO level. First, the concept of MCAT was not well understood among the workforce as the latter kept on referring to ISTs. The frequent questions were, are MCAT similar to ISTs? Are MCATs replacing ISTs? What will happen to the staff, mainly NPOs who were performing the functions to be covered by MCATs? These questions raised more anxiety and stress among staff, particularly those who were designated to move from ISTs to MCAT positions. It also created a sense of job insecurity among the NPOs in the WCOs covered by the MCATs. While the former were not certain about their new roles and responsibilities, their reporting lines and their budgets, the latter feared for redundancy and possible abolition of the national professional positions. To address this challenge, a workshop was organized in 2022 gathering all MCATs staffers and the WRs hosting the MCATs and those to benefit from their support. During this workshop the roles and responsibilities of MCATs were clarified as well as their reporting lines and supervision. Subsequent virtual sessions were organized to address the questions raised by staff across the regions in particular the NPOs to reassure them that MCATs are not conceived to replace the NPO functions but to complement them by providing high level technical expertise.

In addition, the FR further contributed toward a harmonization and standardization of the functions throughout the region which facilitates rotation and mobility of skills and talents across the board. Standard position descriptions were created for similar positions across the 47 WCOs. In this regard, other regions and the WHO Head Quarters adopted this innovative approach and are the outcomes of the FR such as the generic structures and job descriptions for their various staffing needs (24). Thus, the FR contributed in positioning the African Region as a proud leader in Transformation and functional review innovative approaches. However, the funding of MCATs remains a challenge as most of the proposed positions are not filled which raises the issues of the sustainability of the MCATs approach.

### Agile and flexible workforce

The FR took into consideration the changes happening in the global job market and the competition over scarce skills and talents and adjusted accordingly the staffing model of each WCO. As such, the organizational structures of all WCOs highlighted the core positions to be prioritized for filling in line with the limited available resources. Furthermore, the types of appointment offered to staff reflected the nature of the position and the type as well as the duration of the funding. Accordingly, an outreach effort to attract talents and adequate skills to fill vacant positions was launched in collaboration with various actors in this area (7). All managers were required to reach out to their networks to attract the best candidates. This opportunity was also used to attract female candidates and those from under-represented Member States to improve gender balance and geographical representation among WHO workforce in the African region. Once the “must-have” core functions are resourced, managers would fill the gaps through other types of contractors such as the use of temporary workforce, including consultants, volunteers, specialized institutions, and other experts. However, those holding non-staff contracts such as United Nations Volunteers (UNVs), consultants and Special Service Agreement (SSA) holders complain of the precarity of their working conditions, particularly when they stay long under these contractual arrangements. For example, consultants do not have entitlements such as leave, pension and health insurance despite the length of their contracts which can go up to 11 months non-stop. The UNVs are allowed to work under that contractual arrangement for 4 years after which they are separated. Furthermore, all these members of workforce, including holders of Special Services Agreement (SSAs) do not contribute to the UN pension and the non-renewal of their contracts does not provide for any form of separation indemnities and allowances. This put them in a situation of vulnerability and precarity in terms of social security scheme. All these issues contribute to the anxiety, job insecurity and job unsatisfaction for this group of members of the workforce.

Finally, the functional review provided flexibility for expansion when the WCO is responding to an ad-hoc event such as a pandemic outbreak or humanitarian emergency and to shrink after the event is put under control. The agile workforce has proven to be efficient during the response to outbreaks such as Ebola virus, COVID-19, Marburg, and other outbreaks across the region (19, 21). Furthermore, managers have the flexibility to recruit temporary staff to handle an increased workload or to provide the required ad-hoc technical expertise to the host country. Agility and flexibility in workforce management is a critical change and a new way of doing business that the African Region has successfully implemented (24–26). However, the anxiety and stress among the workforce recruited in these circumstances are not negligeable. Whenever there is shortage of funding the workforce with short term appointments and those under non staff contractual modalities are the first to be separated. This was also considered as the source of anxiety and stress among the concerned workforces. The study conducted by Asfawa and Chang (11) indicates that perceived job insecurity was associated with reduced engagement and that this may be moderated by supervisor support. The response of senior management for this situation has always been that all members of our workforce are important, and their contribution is well recognized, valued and respected. It has also been proved that those with these less attractive contractual modalities are given priority when opportunities arise to create and fill fixed term positions. These measures compensate for their less attractive employment conditions and give them hope to accede to a more satisfying and secure contractual arrangement.

### Workforce capacity and capability

One of the best practices of the FR was the process of skill inventory and gap analysis among the workforce at each WCO. This exercise allowed identification of the skills available in each WCO in comparison with what is needed for the WCO to efficiently discharge its mandate. The functional review team identified the skill gaps and made recommendations on how to bridge them. The process of bridging the gaps as recommended by the functional review included upskilling through capacity building programs, exchange programs to stimulate experience from other offices and finally recruiting new talents with the required skill set. Furthermore, existing staff were matched to the functions for which their skills and talent fit. This great innovation in human resources management enabled WCO managers to assign the right people with the right skills to the right functions (24). In addition, the collected information informed the AFRO management to design appropriate capacity building programs such as the mentorship program and the Leadership Pathways to equip managers with the required skills to lead their teams. An organization engaged in a transformation aiming for excellence requires leaders with capacity and capability at all levels of leadership (12, 23). The assessment of workforce capacity and capability is an important exercise to ensure efficient and effective management of activities at WCO levels. All senior staff with managerial roles or supervising teams need the capacity and the capability for optimum performance of individuals and teams under their supervision (22, 25, 26).

However, the process of skill inventory as well as upskilling initiatives raised hopes and expectations among the workforce which some staff considered not fulfilled when no change happened in their career. From the discussions in meetings organized around functional review, staff indicated that they expected that their long experience, adequate educational background and high performance would offer them opportunities for career growth. AFRO.

Management constantly clarified that according to the guidelines set forth to guide FR implementation, the matching of staff to positions would be for same grade or one grade below. Promotions were only possible through a competitive selection process. This principle guaranteed fairness, objectivity, equity and transparency. However, it also created frustration for staff who met the requirements but could not be matched to positions of higher grades than their own. Nevertheless, they were reassured that when these positions are advertised their candidatures would be given priority as internal candidates.

### People and performance management

Cognizant of the difference between having a team with the right skills in the right place and making the team to perform and produce the desired results, the functional review introduced a culture change in people management through managerial and leadership soft skills with an objective of improving performance. Understandably, high performance requires not only technical expertise but also and mainly efficient people management. It has been established that when the workforce is highly engaged, they become more productive, proactive, and motivated (13). However, noting that functional review had created a climate of job insecurity, uncertainty and low morale, raising the level of engagement among workforce was the priority of AFRO management.

A communication strategy aiming at reassuring staff that the purpose of FR is not to lay off staff but to realign functions with the needs and priorities of the host countries contributed to raising staff engagement. Furthermore, the discourse around the benefit of FR in terms of creating opportunities for career progress through the increased number of positions contributed to raising hopes and morale of the workforce. This was also a call for all managers and team leaders to motivate and encourage their team members to voice their concern and to make proposals for culture change. In this regard, AFRO management encouraged innovation and creativity across the region. This was achieved through embracing the spirit of the truism that skilled people should not be told what to do but allowed to express their potential and be guided toward fulfilling their personal goals and driving the organization’s goals. Notably, “human beings have an inherent tendency to seek out novelty and challenges, to extend and exercise their capacities, to explore and learn” (13, 24).

### Good practices in implementing functional review

Implementation of functional review in the 47 WCOs required innovative approaches such as putting up a team dedicated to implement the outcome across the region. The functional review process had taken a long period which had aggravated the status of anxiety among workforce. The implementation of the FR results had to be accelerated so that all staff can know their situation. The Human Resources and Talent management (HRT) team reassigned all staff to positions to which they were matched and adopted a fast-track recruitment approach to speed up the selection process for the remaining vacant positions. This team was also responsible for clarifying the process of implementing the results of the functional review and to respond to individual queries pertaining to specific issues related to contractual arrangements and entitlements. Furthermore, a team of psychologists and stress counselors was put in place to assist staff who experienced anxieties, stress and mental problem.

The filling of more than 450 positions in less than 6 months provides a great inspiration for management to consider the approach for future massive recruitments. The constitution of multiple panels and their use for several selections has created a harmonized, transparent and objective approach in assessing and selecting candidates^1^. The availability and participation of staff in selection panels as hiring managers, independent panel members or representative of staff association, despite their busy schedules was exemplary of staff ownership of the process and is commendable. Therefore, the implementation of the outcome of the functional review encompassed everyone and was not a preserve of the HRT or management alone. Another innovation celebrated as good practice was that the coordination and management of all selection processes including local positions, was conducted at regional level to increase objectivity, transparency and to discharge the managers, particularly the WRs, of the burden of being perceived as subjective or favoring certain staff. However, the coordination was conducted in a manner that managers/WRs were consulted and updated at each stage of the process. As such, collaboration with WRs and Managers across the region facilitated the smooth running of the selection processes making the robust functional review ambition a success.

### Challenges and lessons learned from the functional review implementation

The functional review created negative sentiments among the workforce attributed mainly to the fear of the unknown and job insecurity. The perception that the skill inventory would result in some cases where the current staff profiles were inadequate for the needs and priorities of the country created a climate of uncertainty. Furthermore, the long period that the process lasted created a prolonged state of anxiety. As such, the main challenge was to accommodate and manage these negative sentiments, particularly anxiety, stress and fear which had a toll on staff moral and mental health. Change always brings challenges, resistance, and uncertainty (14). Accordingly, the functional review presented a great test on leadership and people management in times of uncertainty. The fear of the unknown and the negative experiences of previous organizational restructuring and reforms complicated the management of uncertainty at individual and managerial levels. Pertinent questions by staff on whether their positions will be abolished or kept in the new structure; whether the staff will remain in the current duty station or likely to move and to where; Would they be able to keep their children in the good schools like the ones they are currently studying? Such interrogations fueled an environment of uncertainty that Managers at all levels had to respond to as transparently as possible, although they could not provide definite answers. Uncertainty about the future of one’s career is the source of fear, doubt, worry and anxiety which, when not professionally managed, can affect the staff’s wellbeing and the family let alone the working environment.

To address this critical challenge, a communication strategy was necessary to keep staff appraised of the process and outcome. In this regard, frequent update messages from the Regional Director to all staff about the functional review process coupled with Townhall meetings helped to clarify some of the uncertainties and to reassure staff about the objectivity and transparency of the process. Furthermore, WHO management put in place staff counseling and psychosocial support services which helped to accompany staff through the long and challenging FR implementation process.

### Functional review positive outcome

Despite the challenges such as anxiety and stress brought about by the functional review, the latter provided opportunities for career progression among staff. Since 2018, a total number of 473 staff got promoted while many others moved to different functions within and across WCOs through lateral movement (Table 1).

**Table 1:** Upward mobility among WHO/AFR staff since 2018.

Promotions	To grade																	
From grade	G3	G4	G5	G6	G7	NO-A	NO-B	NO-C	NO-D	P1	P2	P3	P4	P5	P6	D1	D2	Total
G1	2																	2
G2	14	2	9															25
G3		3	3	1														7
G4			23	10	4													37
G5				34	8						2	1						45
G6					23	5	6	1		1	6	1						43
G7						9	7				14	7						37
NO-A							2				2	4						8
NO-B								46	3		6	9	6					70
NO-C									5			6	50	2				63
NO-D													4					4
P2												20	2					22
P3													37	1				38
P4														41				41
P5															5	15		20
P6																10		10
D1																	1	1
Total	16	5	35	45	35	14	15	47	8	1	30	48	99	44	5	25	1	437

Furthermore, the functional review implementation introduced some innovations that will continue to be used for the management of such a complex and massive reform. One of these is grouped recruitment and creation of rosters which proved to be a good approach and a commendable good practice to save time and to have a pool of qualified candidates to tap from whenever a recruitment need arises. The creation of roster allowed staff who were not selected for a particular position but were vetted by selection panels as qualified candidates to be hired without further selection process when an opportunity arises within the African region and beyond. In this regard, other regions and HQ continue to contact AFRO to share the candidates from the rosters for their own recruitment. As such, functional review’s positive outcomes profit the region and beyond.

Furthermore, capacity-building activities were and continue to be organized function by function to ensure that the staff who have integrated new teams and/or occupy new functions are well briefed on their work and the deliverables expected of them. Managers at all levels are being trained on leadership and managerial soft skills, in particular, people management and team performance to strengthen their capacity to lead their teams to excellence levels and standards of performance expected of them.

Finally, communication played a significant role in the process of conducting and implementing functional review. The outcome of FR was communicated to all staff and they had an opportunity to comment and ask questions for clarification. Furthermore, the Regional Director communicated regularly with all staff on the progress of functional review at each stage of conception and implementation process (15). As communication is a key for change management, the main message emphasized that the purpose of the functional review was not to reduce staffing but to assign staff in the right places in accordance with their skills and talents. Communication throughout the process and accompanying measures such as staff counseling and psychosocial support services played a determining role in overcoming the challenges and making the FR process a success.

### Lessons learned and way forward

The main lesson learned was that a robust transformation carries challenges such as uncertainty and anxiety among the workforce. As such, beyond the FR implementation, the remaining critical work is to remove the uncertainty and anxiety through a communication strategy and a robust system of stress management and mental health investment. Furthermore, staff engagement and motivation is of paramount importance for individual and team performance. As such, the Human Resources and Talent management Unit (HRT) is required to work with Managers to invest in activities aiming to improve staff engagement and motivation. Management should invest more in the already launched staff development initiatives such as induction, mentorship, team performance program and leadership pathways. Effective communication, team spirit and collaboration among staff within and across budget centers will be a key factor for maintaining and improving the gains of functional review. AFRO senior management commits to accompany individual staff, Managers and teams to perform to their highest potential by creating a respectful and empowering work environment and giving a platform to staff to air their views. Investment in stress management and staff wellbeing will go a long way in creating a workforce engaged and motivated to discharge the mandate of WHO in the African region. Regular satisfaction surveys will inspire and guide management on areas to improve.

## Conclusion

The FR proposed an innovative approach consisting of planning focused on the needs and priorities of the country as opposed to the traditional budget-based planning. In the spirit of the FR, each WCO designed its structure aiming at addressing the prevailing health situation, needs and priorities of the host country. In so doing, the FR did not only offer an innovative way of presenting the planning, budgeting and implementation of activities at WCO level but also provided a possibility to shift from a budget-based planning approach to the needs and priority-based approach.

By providing WCOs with ideal structures and relevant workforce planning aligned with the health situation, needs and priorities, WHO at country level is now able to approach donors and showcase their added value focusing on where they can make an impact if funding was channeled to the identified needs and priorities (10). The structures derived from the FR highlight the critical functions that WCO must have to deliver effectively and efficiently its mandate. As such, through the structures provided by the FR, WCOs found a tool reflecting the views of stakeholders and partners which can be used for resource mobilization and advocacy. Where the mobilized funding does not cover the entire functions in the structure, WCOs prioritize the critical functions and continue to mobilize resources with the spirit of staffing the remaining positions as and when funds are mobilized.

Furthermore, the FR introduced an innovative way of speedy and timely talent acquisition through grouped recruitments and creation of roster for future rapid filling of vacant positions.

However, the FR came with some challenges and negative effects on the workforce. It created a climate of uncertainty, a feeling of job insecurity which were a source of anxiety and stress among the workforce. Furthermore, the FR and its implementation took a long time which prolonged the period of uncertainty and anxiety among workforce. Moreover, its implementation happened during the acute outbreak of COVID-19 pandemic which added a level of anxiety and stress among the workforce. The management responded to all these challenges through frequent communications to explain the processes and to update staff on the status of implementation. Accompanying measures such as preparation of staff through briefings on CV writing and how to do competency-based interviews were appreciated by the workforce. Furthermore, the introduction of psychologists and stress counselors to assist staff in need was a great move in people management in times of uncertainty.

## Footnotes

^1^FRW implementation guidelines (Internal document).
